# Acne on the Valve: Two Intriguing Cases of Cutibacterium Acnes Endocarditis

**DOI:** 10.7759/cureus.8532

**Published:** 2020-06-09

**Authors:** Samra Haroon Lodhi, Ayesha Abbasi, Taha Ahmed, Albert Chan

**Affiliations:** 1 Internal Medicine, King Edward Medical University, Lahore, PAK; 2 Internal Medicine, Mayo Hospital, Lahore, PAK; 3 Internal Medicine, Cleveland Clinic Foundation, Cleveland, USA; 4 Cardiovascular Medicine, Cleveland Clinic Fairview Hospital, Cleveland, USA

**Keywords:** cutibacterium acnes, endocarditis, rocking valve, prosthetic valves, paravalvular leak, vegetations, aortic root abscess

## Abstract

Cutibacterium acnes is a skin commensal which is most often regarded as a contaminant when detected on blood cultures. In rare instances, it may be the causative pathogen in severe systemic illnesses. Subacute endocarditis, especially of prosthetic valves and devices, is an important grave pathology caused by Cutibacterium acnes. Herein we report two cases of prosthetic valve endocarditis with varied presentations as valve dehiscence with a “rocking” prosthetic valve apparatus in one encounter and as a septic embolic stroke in the second encounter. Although a rare cause of endocarditis, it becomes an especially important entity in patients with prosthetic devices and should be high in the list of differentials.

## Introduction

Prosthetic cardiac valves have been labeled as a predisposing cardiac condition for infectious endocarditis in the guidelines provided by the American Heart Association [1]. *Cutibacterium acnes* (*C. acnes*) is a micro-aerophilic, non-spore-forming, gram-positive coccobacillus which constitutes a part of the normal skin flora. Although known as a causative agent of acne, it has been associated with various severe infections including endocarditis, osteomyelitis, arthritis, spondylodiscitis, endophthalmitis, post-craniotomy, and ventriculoperitoneal shunt infections [2]. The predominant predisposing conditions include previous surgical interventions and the presence of foreign bodies such as prosthetic valves. Thus, *C. acnes* is often regarded as an under-estimated pathogen in late postoperative infections [2]. Herein we report two cases of prosthetic valve endocarditis (PVE) caused by *C. acnes*.

## Case presentation

Case 1

A 77-year-old hypertensive male presented to the ED with dyspnea on exertion, fatigue, and pedal edema for the last three weeks. He further admitted to experiencing night sweats, daytime clamminess, and episodes of transient visual loss for the last two months. The patient reported a 20-pound weight loss which he attributed to a low salt diet. He denied fever, loss of consciousness, chest or abdominal pain, or prior shortness of breath before this presentation. Medical history was significant for calcific aortic stenosis with aortic root ectasia status post-surgical valve replacement (23 mm Carpentier-Edwards pericardial aortic valve [Baxter Healthcare Corporation, Edwards CVS Division, Irvine, California, US] with resection and replacement of ascending aorta with a 28 mm Hemashield graft [Boston Scientific, Natick, Massachusetts, US]) seven years prior, paroxysmal atrial fibrillation, and chronic first-degree AV block. On a recent visit to his physician’s office three months ago, an increase in the intensity of combined systolic and diastolic murmur was noticed. Subsequently, a transthoracic echocardiogram revealed moderate central aortic regurgitation (AR) with leaflet degeneration.

On presentation, the patient had a temperature of 98.3° F, blood pressure of 86/42 mmHg with a heart rate of 60 beats per minute, and a respiratory rate of 26 breaths per minute. His hypotension improved with fluid resuscitation in the ED and he was sent to the regular medical floor. Physical examination revealed warm, well-perfused extremities, elevated jugular venous pulse, loud III/VI crescendo-decrescendo murmur at the second right intercostal space along with a diastolic murmur at the left sternal border, bibasilar crackles on lung auscultation and peripheral edema. Electrocardiogram (ECG) showed atrial flutter with a variable block. Troponin T was elevated at 0.116 ng/mL (normal range 0-0.029 ng/mL) and brain natriuretic peptide was high at 5202 pg/mL (normal < 99 pg/mL). Complete blood count revealed an elevated white blood cell count of 15,990/UL, and chronic anemia with hemoglobin around the baseline of 10.9 g/dL. Given the high index of suspicion for PVE, two sets of blood cultures were drawn. Subsequently, a trans-esophageal echocardiogram (TEE) revealed an aortic root abscess and large vegetation with “rocking motion” of the aortic valve prosthesis, consistent with dehiscence as well as a severe paravalvular leak with preserved left ventricular size and function (Videos 1-3).

**Video 1:** Trans-esophageal echocardiogram mid-esophageal short axis-view Bulky vegetation on the aortic valve (yellow arrow) and severe aortic paravalvular leak (blue arrow)

**Video 2:** Trans-esophageal echocardiogram mid-esophageal long-axis view "Rocking motion" of the aortic valve prosthesis during systole and diastole (blue arrow)

**Video 3:** Trans-esophageal echocardiogram mid-esophageal long-axis view with color Doppler Prosthetic aortic valve dehiscence with paravalvular leak (blue arrow)

The patient was transferred to the cardiac intensive care unit for dynamic monitoring for the risk of progression of the atrioventricular (AV) block. The cardiothoracic surgery department was consulted and it was deemed a surgical emergency. A cerebral angiogram revealed no evidence of mycotic aneurysms and the patient underwent a redo surgical aortic valve replacement. Intra-procedural observations included an extremely infected prosthetic valve with multiple vegetations on both surfaces causing near obstruction and a semicircular root abscess that completely destroyed the aortic mitral membrane. The abscess was cleaned and the material was sent for culture. Subsequently, the aortic valve and homograft were replaced with new prostheses with no significant paravalvular leak or wall motion abnormalities in the intra-procedural echocardiogram. Empiric treatment with broad-spectrum antibiotics was maintained.

Blood cultures grew C. acnes nine days after they were drawn. Moreover, cultures of the explanted prosthesis as well as the aortic root abscess material grew C. acnes. The patient had a peripherally inserted central catheter (PICC) placed for community-based parenteral antimicrobial therapy with intravenous ceftriaxone.

The patient successfully completed cardiac rehabilitation and the antibiotic course and PICC was removed at the follow-up visit with no recurrence of symptoms. The follow-up electrocardiogram showed sinus rhythm with chronic first-degree AV block and echocardiogram at 3 months showed stable aortic valve gradients and no aortic valve regurgitation (Video 4).

**Video 4:** Follow up trans-esophageal echocardiogram in the mid-esophageal short-axis view with color Doppler Normally functioning aortic valve prosthesis

Case #2

A 49-year-old male presented to the ED with sudden onset diplopia one hour prior to arrival. He reported experiencing night sweats and chills for the preceding five weeks. His cardiovascular history was significant for aortic valve replacement surgery with 21 mm Trifecta St. Jude bio-prosthesis (St. Jude Medical, Inc., St. Paul, Minnesota, US) 15 months earlier for bicuspid aortic valve stenosis. Physical examination revealed stable vital signs and left inferior visual field defect was appreciated with a normal funduscopic exam. The patient was alert and oriented. The findings from the examination of the lungs, abdomen, extremities, and neurological functioning were unremarkable. The CT scan of the head showed no acute intracranial abnormality. Initial workup including complete blood count and metabolic panel were satisfactory. Chest x-ray and urine analysis were normal.

Electrocardiogram revealed sinus rhythm with no acute ST-T wave changes. Brain magnetic resonance imaging (MRI) showed evidence of stroke of the occipital cortex involving the distribution of the posterior cerebral artery. Thrombolysis was not administered due to a low NIH (National Institute of Health) Stroke Scale Score. In the face of clinical suspicion of infective endocarditis with septic emboli, two sets of blood cultures were drawn and the patient was commenced on intravenous vancomycin. Trans-esophageal echocardiogram demonstrated small vegetation on the prosthetic valve (Video 5).

**Video 5:** Trans-esophageal echocardiogram mid-esophageal short-axis view Small vegetation on the prosthetic aortic valve (blue arrow)

Cardiothoracic surgery was consulted. Within 48 hours of the hospital stay his vision returned to the baseline. Blood cultures remained negative but he was kept on empiric broad-spectrum antibiotics. The patient was discharged on day 4 with a diagnosis of culture-negative PVE on intravenous ceftriaxone to be administered via a peripherally inserted central catheter (PICC).

The patient underwent redo aortic valve replacement 15 days later. Intra-procedural findings were significant for findings consistent with 1 - 1.5 cm fibrinous gelatinous vegetation on the aortic side of the left cusp and thickening with pannus formation under the right and the left cusps on the ventricular side. There was a circumferential abscess cavity within which the valve was present. The valve and vegetation were sent for testing and a new prosthetic valve was placed. The abscess cavity was drained.

16S ribosomal RNA testing on the valve vegetation material confirmed *Cutibacterium acnes*. The blood culture drawn during the index admission also grew *C. acnes*, almost 50 days later. Based on the diagnosis of *C. acnes* prosthetic valve endocarditis, antibiotics were switched to penicillin G and gentamicin after reviewing the minimal inhibitory concentrations (MICs). The patient finished this prolonged treatment course for two months successfully with no recurrence of symptoms at the six-month follow-up.

## Discussion

*Cutibacterium*, also known as *Propionibacterium*, is a ubiquitous genus of bacteria that exist as part of the normal skin flora and mucosal surfaces (the conjunctiva, external ear, nasopharynx, oral cavity, and genitourinary tract) as well as a common environmental surface contaminant [3,4]. These are associated with three types of infections: acne on the skin, surgical site infections, and invasive deep-seated infections, particularly in the setting of endovascular devices such as pacemakers, defibrillators, shunts, and prosthetic devices [5-7]. In general, PVE has an incidence of 0.3 and 1.2 cases per patient-year, affecting about 1-6% of patients with a cardiac valve prosthesis. *Cutibacterium *constitutes a rare, yet frequently underrecognized cause of PVE [1]. *C. acnes* has been shown to cause both native and prosthetic valve endocarditis; however, the vast majority of cases are associated with aortic valve prostheses [8,9]. Despite its presumed low virulence, the ability to cause deep-seated infections is attributed to its ability to form biofilms [10]. The mortality rate of Propionibacterium acnes prosthetic valve endocarditis (PAPVE) is 15-27%, which is much lower than the endocarditis caused by other organisms. This might be attributed to the indolent nature of this organism. However, by virtue of its slow-growing course, Cutibacterium leads to extensive destruction of valvular and perivalvular tissues before the diagnosis is made and frequently requires surgical intervention for a cure [5,9,11].

The classical stigmata of infective endocarditis are usually lacking in C. acnes PVE due to the indolent nature and slow growth of the organism [11,12]. One case series reported asymptomatic prosthetic valve dysfunction in 31%, heart failure in 19%, coronary syndrome in 12.5%, fever in 25%, and neurological deficits in 19% at presentation [13]. Another case series reported peripheral emboli in 16%, brain emboli in 10%, myocardial abscess in 36%, and valvular insufficiency in 52% cases as the various complications resulting from PAPVE [14]. These cases included two major presentations including heart failure due to severe valvular insufficiency and brain emboli. An interesting finding in one was the finding of a “rocking" valve due to dehiscence.

Among demographic factors, the most peculiar finding is the male preponderance in PAPVE, with 100% cases reported to be males in one of the largest case series conducted to date [15]. The exact reasons are unclear, although the greater number of sebaceous glands and a higher incidence of cardiac implantable devices in males have been implicated in some studies [14]. Our study conforms to the above series, with both the patients being male. The time interval between the index surgery and the presentation of a patient with PAPVE is between five to 135 months [15]. Our first case had an interval of 84 months after valve replacement and second about 18 months from index surgery, falling within this time frame. Other case series report a mean interval of 14 years following the valve replacement surgery [5]. Shorter time periods may be attributed to inoculation during surgery followed by slow, indolent destruction of valvular tissues, whereas longer periods(>11 years or >23 years) most likely result from a distant bacterial focus leading to secondary metastasis to the valve [5,16]. Interestingly, the risk of invasive *Cutibacterium *infection is not reduced with perioperative skin preparation.

*C. acnes* infections present a unique diagnostic dilemma. Due to its ubiquitous nature, it is frequently regarded as a culture contaminant especially when obtained from a single culture. Its detection requires both aerobic and anaerobic blood and tissue cultures incubated for up to two weeks, whereas most microbiology laboratories incubate cultures for only one week. Modern diagnostic tests such as valve sequencing are more sensitive and may lead to greater rates of detection of PAPVE [16]. This was demonstrated in our second case where the cultures did not grow *Propionibacterium acnes* until at least 50 days from index admission. 16S ribosomal RNA testing on biopsy material aided the diagnosis. Furthermore, positive *Cutibacterium* cultures and histopathological evidence of gram-positive coccobacilli from the infected valve can also support the diagnosis, especially in cases of blood culture-negative( one-third cases) or single blood culture positive PAPVE [12]. The echocardiogram shows vegetations in up to 60-70% cases [5].

Evidence of any prosthetic valve "rocking motion" by echocardiography is indicative of significant dehiscence [17]. In our study, a classical “rocking” motion of the aortic valve prosthesis was detected on the echocardiogram. The valve moved to the aorta in systole, where it prolapsed to the left ventricle in diastole, resulting in a severe paravalvular jet of aortic insufficiency (AI). These findings were corroborated during surgery. Of its many causes, when valve dehiscence is consequent to infective endocarditis, the mortality of this life-threatening situation is compounded [18]. The degree of aortic insufficiency severity can be predicted by the degree of valvular dehiscence.

As far as the treatment is concerned, most patients with PAPVE require a combination of antibiotics and valve replacement surgery [5,16]. The first line antibiotic is parenteral penicillin (2-3 million units six times daily), with or without the addition of an aminoglycoside (gentamicin 3 mg/kg intramuscular or intravenous every 24 hours in two to three divided doses), for a minimum period of six weeks [11]. Many studies have also reported the efficacy of rifampin in treating PAPVE due to its ability to penetrate biofilms [19]. The indications for valve surgery include congestive heart failure, cardiac abscess, or valve dehiscence. The reported rate of surgical intervention for PAPVE is much higher than that for infective endocarditis caused by other organisms [5,12]. Following valve replacement for prosthetic valve endocarditis, the Task Force on Infective Endocarditis of the European Society of Cardiology recommends another six-week course of antimicrobial treatment if the intraoperative valve culture is positive [20]. Overall, the optimal management of PAPVE yields a favorable prognosis for these patients [5].

## Conclusions

*C. acnes* should be considered a very serious pathogen in patients with foreign body placements and in such circumstances should not be disregarded as a contaminant. Although slow-growing with an indolent clinical course, this bacterium commonly causes infiltrative disease with abscess formation, which may cause serious consequences including valvular dehiscence, heart failure, conduction abnormalities, and embolic strokes. Patients with late-onset prosthesis-related endocarditis often present with vague symptoms and no fever, making diagnosis difficult. The diagnosis is further obscured by the prolonged times needed to grow the bacterium on aerobic and anaerobic cultures. It is often necessary to obtain a trans-esophageal echocardiogram and multiple blood cultures, along with advanced PCR testing. Management with appropriate antibiotics serves to provide clinical control, but surgery with valve replacement is critical to achieving a complete cure.
